# Supplementary material for: Membrane Localization of HspA1A, a Stress Inducible 70-kDa Heat-Shock Protein, Depends on Its Interaction with Intracellular Phosphatidylserine
Source: Biomolecules. 2019 Apr 17;9(4):152. doi: 10.3390/biom9040152 (PMC6523125; doi:10.3390/biom9040152)
Supplement: Supplementary file 1 [file biomolecules-09-00152-s001.pdf]

Membrane localization of HspA1A, a stress inducible 70-kDa heat shock protein, depends on its interaction with intracellular phosphatidylserine

Andrei D. Bilog[1#], Larissa Smulders[1#], Ryan Oliverio[1], Cedra Labanieh[1], Julianne Zapanta[1], Robert V. Stahelin[2], Nikolas Nikolaidis[1,*]

[1] Department of Biological Science, Center for Applied Biotechnology Studies, and Center for Computational and Applied Mathematics, College of Natural Sciences and Mathematics, California State University Fullerton, Fullerton, CA 92834-6850; abilog@fullerton.edu (A.B); l.smulders@csu.fullerton.edu (L.S.); rolive1011@csu.fullerton.edu (R.O.); cedralabanieh@csu.fullerton.edu (C. L.); Julianne Zapanta <jzap@csu.fullerton.edu (J. Z.)

[2] Department of Medicinal Chemistry and Molecular Pharmacology and the Purdue University Cancer Center, Purdue University, West Lafayette, IN, 47907, USA; rstaheli@purdue.edu (R.V.S)

# these authors contributed equally

* Correspondence: e-mail: nnikolaidis@fullerton.edu ; Tel.: 001-657-278-4526

Supplementary Information

Supplementary Figures: 1-8

Supplementary Figure Legends

Figure S1. The cells showed in Fig. 1 expressing empty EGFP or GFP-HspA1A are presented in color. The cells were grown under normal conditions (37°C) or heat-shocked for 1h at 42°C and allowed to recover for 8 hours. The images showing the +ve PDM (see materials and methods) values, where included to show co-localization. Scale bar=10μm.

Figure S2. (A) Representative images of HeLa, Hek293, and HepG2 cells expressing GFP-HspA1A alone, or GFP-HspA1A and mCherry-LactC2, together showing that the HspA1A-PM localization is inhibited in the presence of LactC2. (B) Representative images of HeLa cells expressing GFP-HspA1A show that sphingosine (SP) treatment does not affect the PM localization of HspA1A. (C) Representative images of HeLa cells overexpressing the polybasic charge sensor R-Pre-GFP. (D) Representative images of HeLa cells in which HspA1A-RFP was co-transfected with the PI(4,5)P_2 biosensor PLCδ-PH-GFP. The images showing the +ve PDM (see materials and methods) values, where included to show co-localization. Scale bar=10μm.

Figure S3. Quantification of the corrected total cell fluorescence (CTCF) as a ratio between the total GFP-HspA1A fluorescence of the plasma membrane and the rest of the cell. Center lines show the medians; box limits indicate the 25th and 75th percentiles as determined by R software; whiskers extend 1.5 times the interquartile range from the 25th and 75th percentiles; crosses represent sample means. The experiment was repeated three times and the number of cells used to generate the graphs was n=30. The P values of the student t-test are given below. For Hek293 cells, these values were: HspA1A-37/HspA1A-42<0.0001; HspA1A-37-LactC2/HspA1A-42-LactC2=0.1271; HspA1A-42/HspA1A-42-LactC2<0.0001; HspA1A-37/HspA1A-37-LactC2=0.0011. For HepG2 cells, the P values were: HspA1A-37/HspA1A-42<0.0001; HspA1A-37-LactC2/HspA1A-42-LactC2<0.0001; HspA1A-42/HspA1A-42-LactC2<0.0001; HspA1A-37/HspA1A-37-LactC2<0.0001.

Figure S4. Quantification of the cells expressing active caspase 3/7 using the Caspase-Glo® 3/7 Assay (Promega). (A) The total luminescence (after subtraction of the background values) expressed as relative light units (RLU) was plotted for the different treatments. Cells treated with 1μM staurosporine (STS) for 4 hours at 37 ^{o}C were used as a control. The Y-axis is shown in log scale for clarity. (B) The raw data presented in A were divided by the luminescence values produced by STS treatment and are shown as percent of apoptotic cells. For both boxplots center lines show the medians; box limits indicate the 25th and 75th percentiles as determined by R software; whiskers extend 1.5 times the interquartile range from the 25th and 75th percentiles; crosses represent sample means. The experiment was repeated three times.

Figure S5. (A) Representative images of HeLa cells expressing GFP-HspA1A and mCherry-LactC2 (left panels) or Lact-C2-GFP (with WGA-AF555 PM stain; right panels) showing that the localization of Lact-C2 is not affected by heat-shock or by the presence of HspA1A. The images showing the +ve PDM (see materials and methods) values, where included to show co-localization. Scale bar=10μm. **(B)** Quantification of the corrected total cell fluorescence (CTCF) as a ratio between the total Lact-C2 fluorescence of the plasma membrane and the rest of the cell. Center lines show the medians; box limits indicate the 25th and 75th percentiles as determined by R software; whiskers extend 1.5 times the interquartile range from the 25th and 75th percentiles; crosses represent sample means. The experiment was repeated three times and the number of cells used to generate the graphs was (from left to right) n=14, 16, 15, 17. **(C)** Cells surface biotinylation of cells expressing Lact-C2-GFP. The antibodies used were: the OmicsLink™ Anti-GFP Tag Antibody Mouse Monoclonal IgG1 [(CGAB-GFP-0050); 1:1000- detects a protein band of approximately 45kDa (Lact-C2+GFP)]; the beta actin antibody, Clone: 13E5, Cell Signaling [(Rabbit mAb #4970) 1:1000- detects a protein band of approximately 42kDa]; and the Na+/K+ ATPase α (ATP1A1) antibody RabMAb® [(EP1845Y); (2047-1). All blots were incubated with the antibodies overnight

(~16 hours) at 4°C with constant rotating. M: molecular size marker (Fisher BioReagents™ EZ-Run™ Prestained Rec Protein Ladder; approximate sizes shown on the left side of the blots).

Figure S6. (A) Representative images of HeLa cells expressing RFP-HspA1A and GFP-PLCδ-PH (top two panels) or GFP-PLCδ-PH (with WGA-AF555 PM stain; bottom two panels) showing that the localization of GFP-PLCδ-PH is not affected by heat-shock or by the presence of HspA1A. The images showing the +ve PDM (see materials and methods) values, where included to show co-localization. Scale bar=10μm. **(B)** Quantification of the corrected total cell fluorescence (CTCF) as a ratio between the total GFP-PLCδ-PH fluorescence of the plasma membrane and the rest of the cell. Center lines show the medians; box limits indicate the 25th and 75th percentiles as determined by R software; whiskers extend 1.5 times the interquartile range from the 25th and 75th percentiles; crosses represent sample means. The experiment was repeated three times and the number of cells used to generate the graphs was (from left to right) n=16, 19, 15, 14. **(C)** Isolation of total PM proteins of cells expressing HspA1A-RFP alone, and HspA1A-RFP together with GFP-PLCδ-PH. Nitrocellulose membranes used were stained with the Reversible Protein Stain Kit (Pierce) followed by western analysis. The antibodies used (in order of use) were: RFP Tag Monoclonal Antibody (RF5R) [(MA5-15257); 1:1000- detects a protein band of approximately 98kDa (HspA1A+GFP)]; OmicsLink™ Anti-GFP Tag Antibody Mouse Monoclonal IgG1 [(CGAB-GFP-0050); 1:1000- detects a protein band of approximately 55kDa (PLCδ-PH+GFP)]; and Na+/K+ ATPase α (ATP1A1) antibody RabMAb® [(EP1845Y); (2047-1); 1:1000- detects a protein band of approximately 112kDa; note that there is no band detected in the cytosolic fractions probed with the anti-ATP1A1; the visible band in the cytosolic fractions corresponds to the product of the anti-RFP detecting the HspA1A-RFP protein, which was produced in the first step of this experiment]. All blots were incubated with the antibodies overnight (~16 hours) at 4°C with constant rotating. M: molecular size marker (BioRad Dual Color Protein Ladder; approximate sizes shown on the left side of the blots).

Figure S7. The cells showed in Fig. 4 stained with TopFluor-PS (green) and expressing RFP-HspA1A (red) are presented in color to better visualize the co-localization [merged images and PDM (with PDM scale bar and positive (+ve) PDM values (see materials and methods for details)]. Scale bar=10μm.

Figure S8. Cells surface biotinylation reveals that HspA1A's PM localization depends on its binding to PS and it is not affected by the total membrane charge. (A) Representative Western blots showing the total and biotinylated fractions of HEK293 cell lysates transfected with HspA1A-GFP and RFP-C1 vector, and HspA1A-GFP and Lact-C2-mCherry. The antibody used was the OmicsLink™ Anti-GFP Tag Antibody Mouse Monoclonal IgG1 [(CGAB-GFP-0050); 1:1000]; (B) The nitrocellulose membranes used in (A) were stained with the Reversible Protein Stain Kit (Pierce) and were also blotted with the control antibodies listed below. The antibodies used were the Beta Actin, Clone: 13E5, Cell Signaling [(Rabbit mAb #4970) 1:1000]; Na+/K+ ATPase α (ATP1A1) antibody RabMAb® [(EP1845Y); (2047-1); 1:1000]; and RFP Tag Monoclonal Antibody (RF5R) [(MA5-15257); 1:1000)]. M: molecular size marker (Fisher BioReagents™ EZ-Run™ Prestained Rec Protein Ladder; approximate sizes shown on the left side of the blots). (C) Quantification of the antibody detected signals of the HspA1A (GFP tagged) in the presence or absence of Lact-C2, as well as the Lact-C2 (mCherry tagged) presented as a ratio between the biotinylated (PM) fraction and the total cell lysate. Densitometry values are averages of three independent experiments (n=3). Center lines show the medians; whiskers extend 1.5 times the interquartile range from the 25th and 75th percentiles; crosses represent sample means. The P values of the student t-test were: HspA1A-37-SP/HspA1A-42-SP=0.0009; HspA1A-37-SP/HspA1A-37-LactC2-SP=0.8548; HspA1A-42-SP/HspA1A-42-SP=0.0005; HspA1A-37/HspA1A-37-SP= 0.2742; HspA1A-42/ HspA1A-42-SP=0.1617.

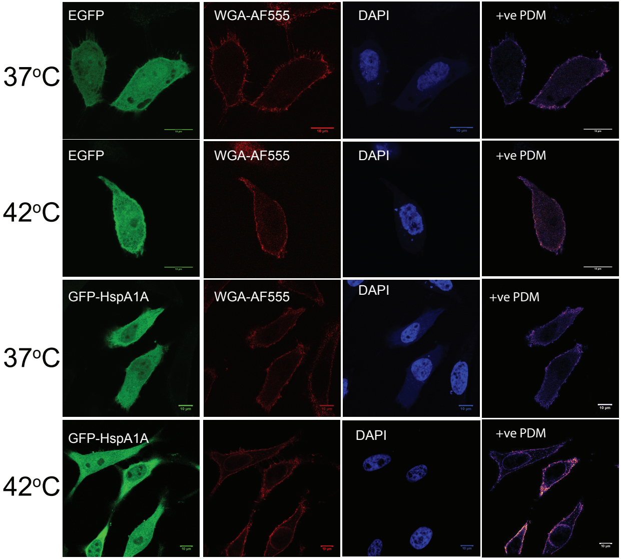

Supplementary Fig. 1

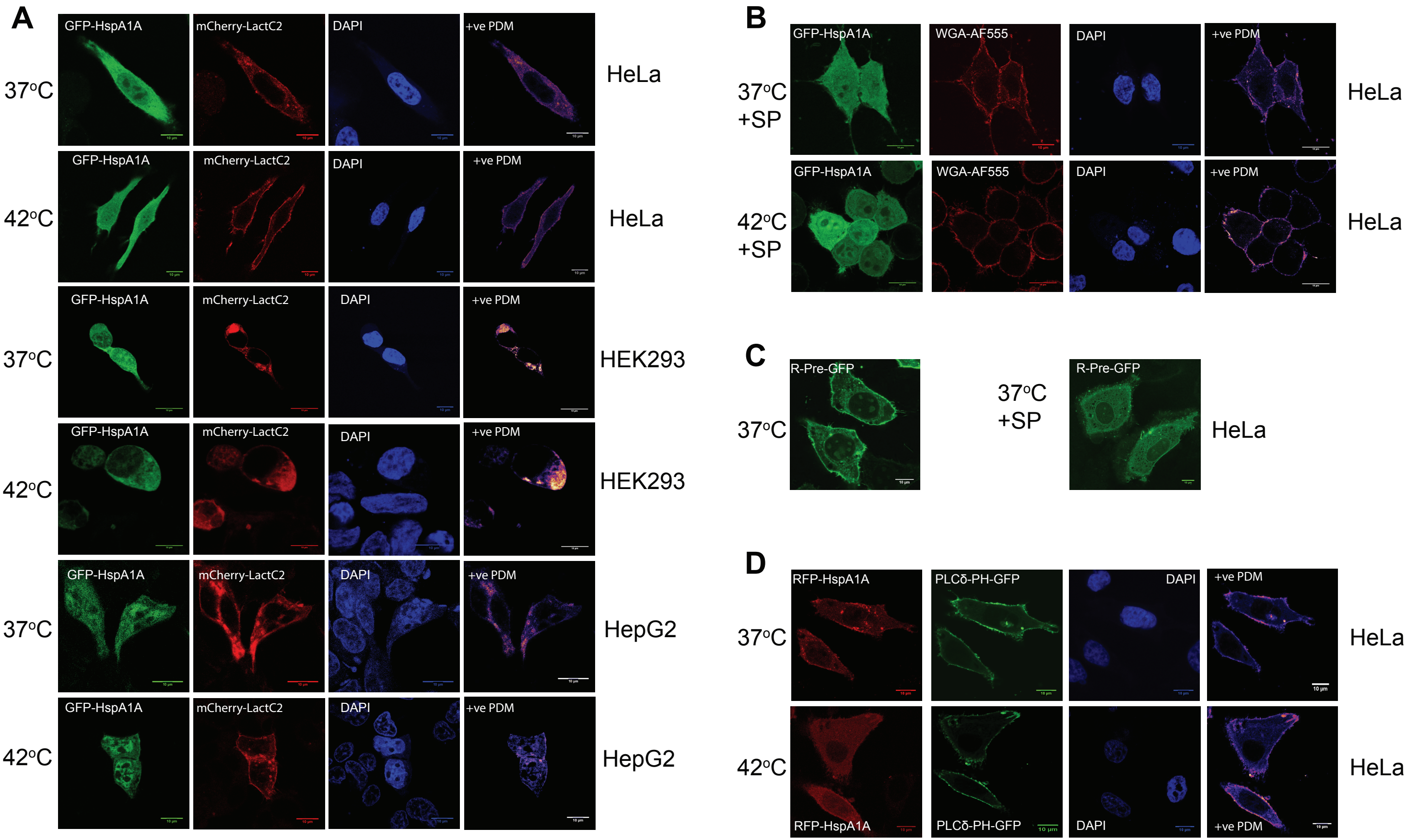

Supplementary Fig. 2

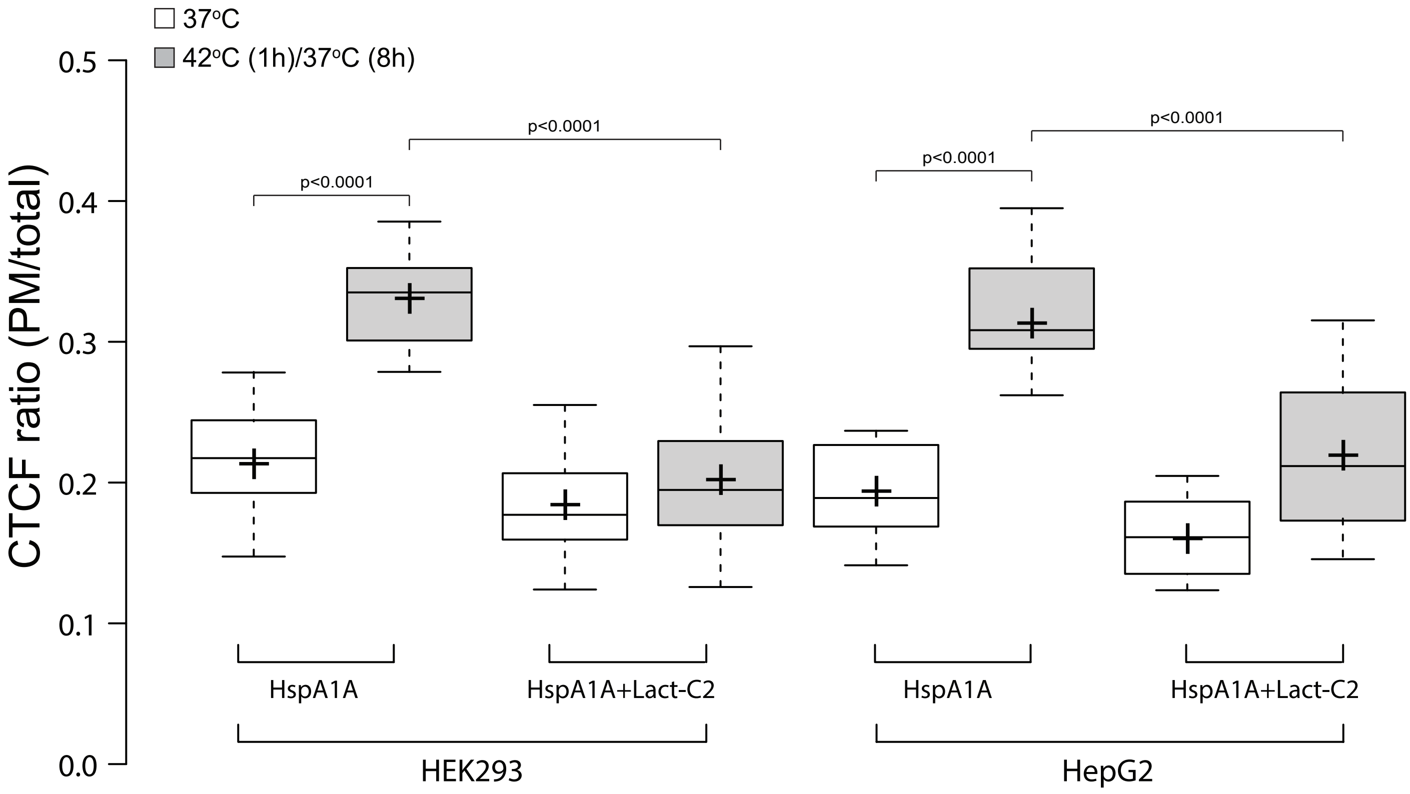

Supplementary Fig. 3

A

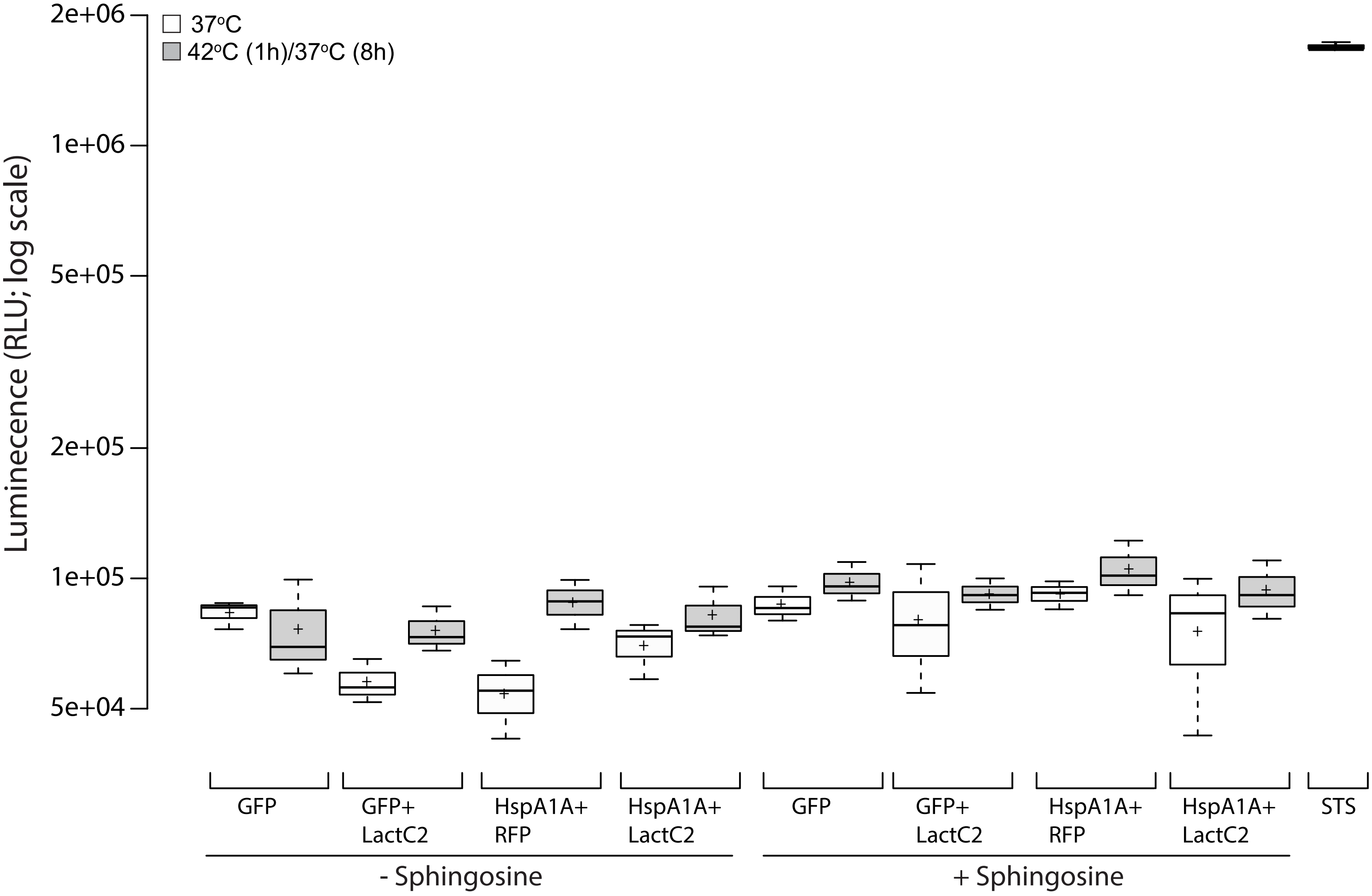

B

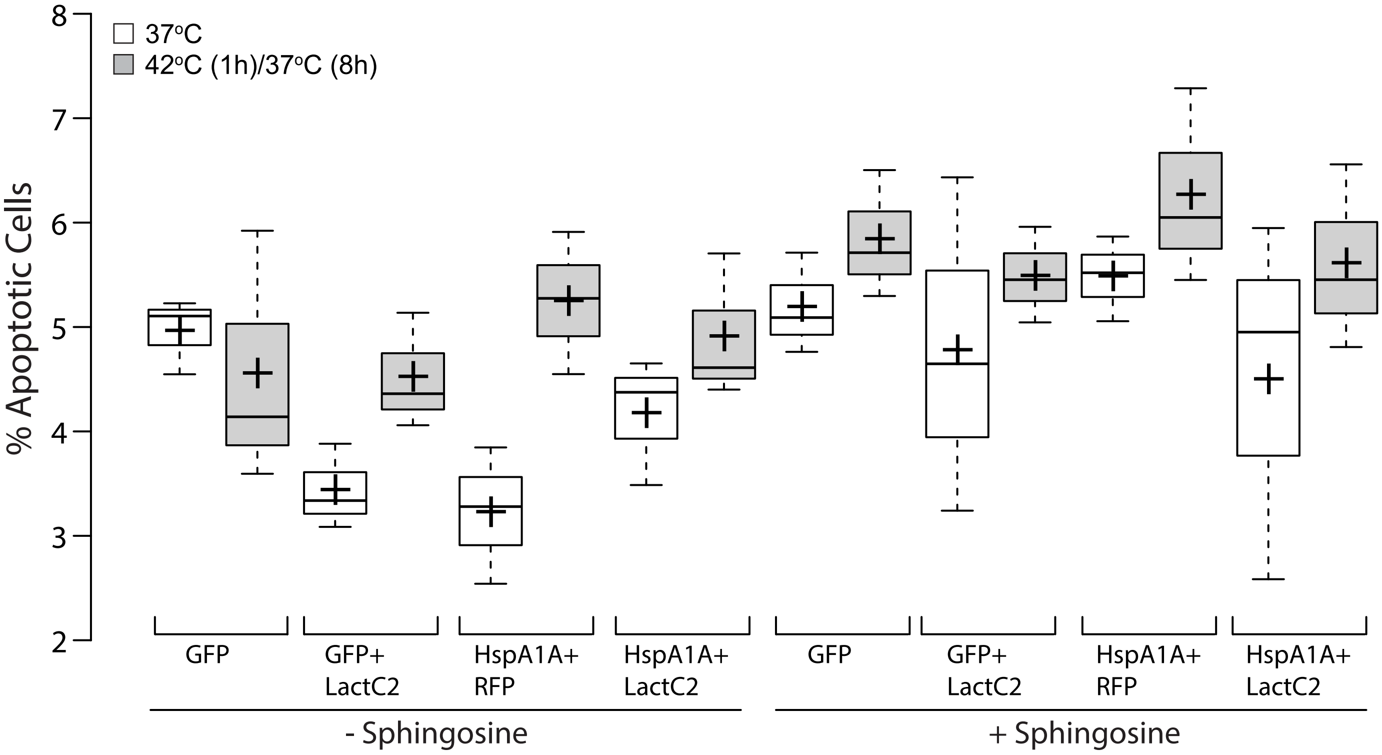

Supplementary Fig. 4

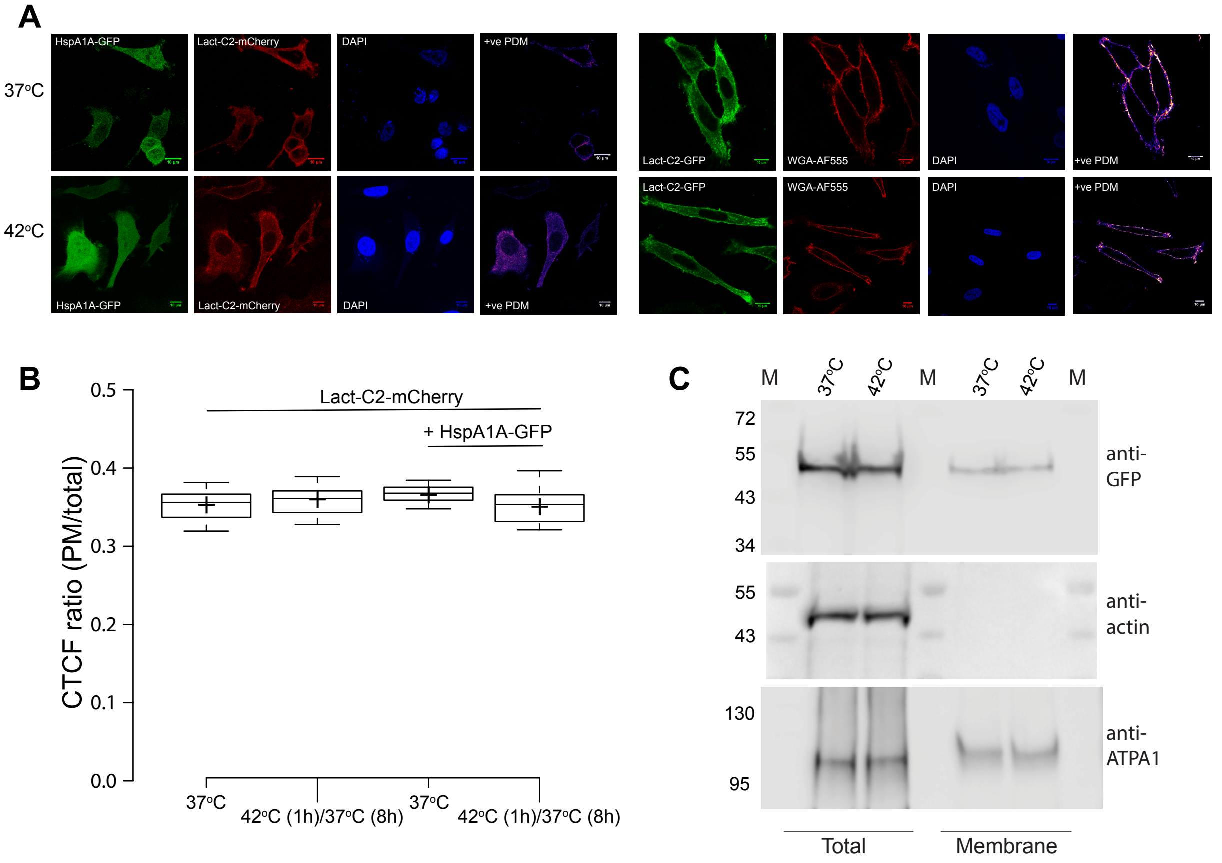

Supplementary Fig. 5

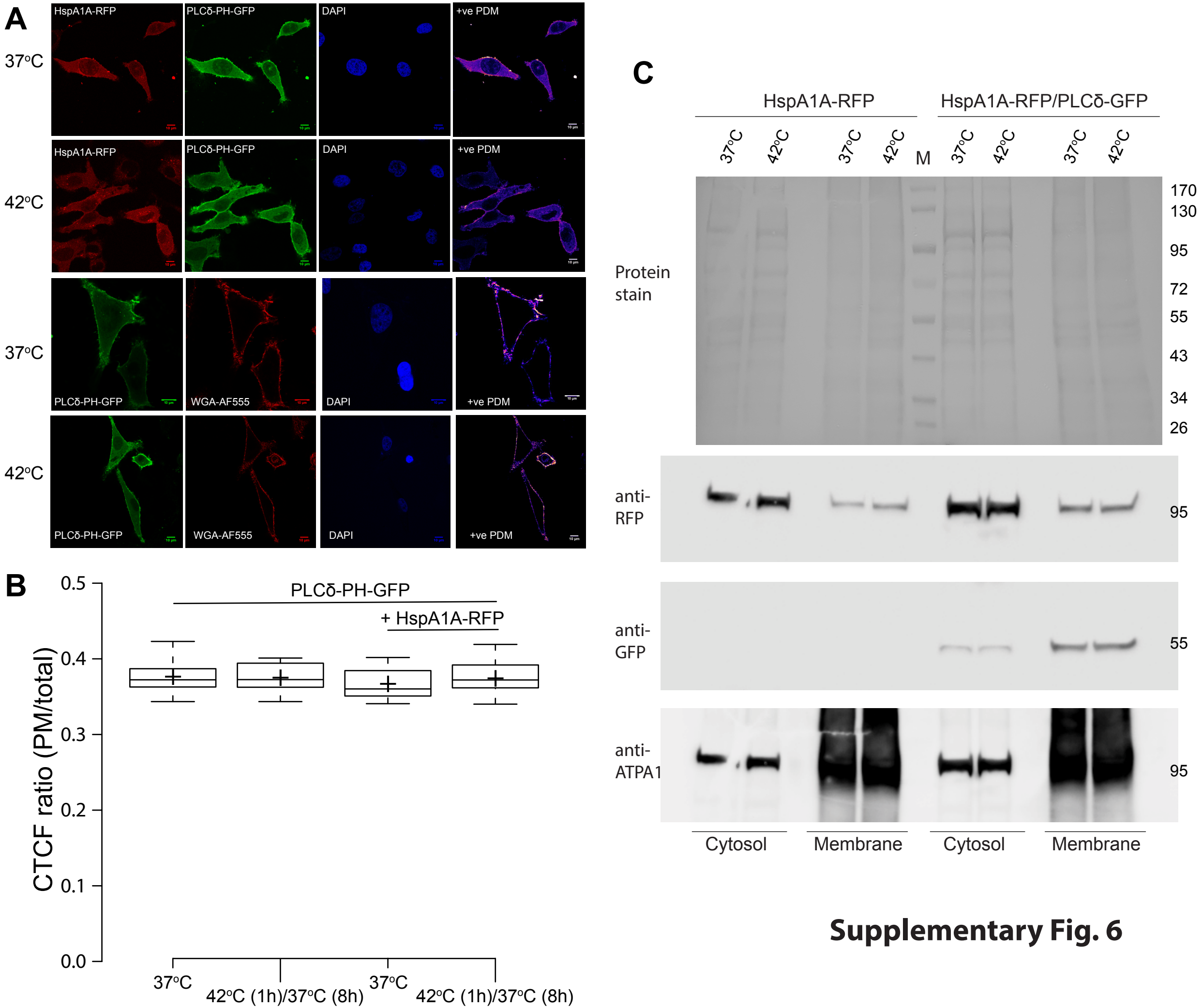

Supplementary Fig. 6

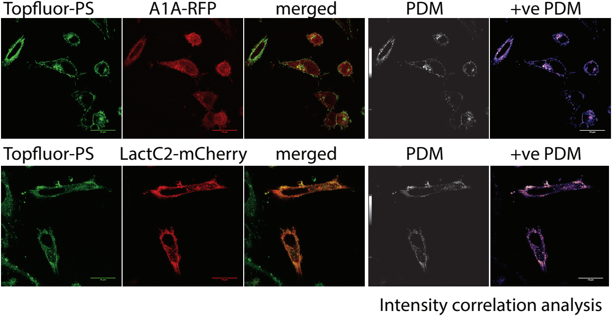

Supplementary Fig. 7

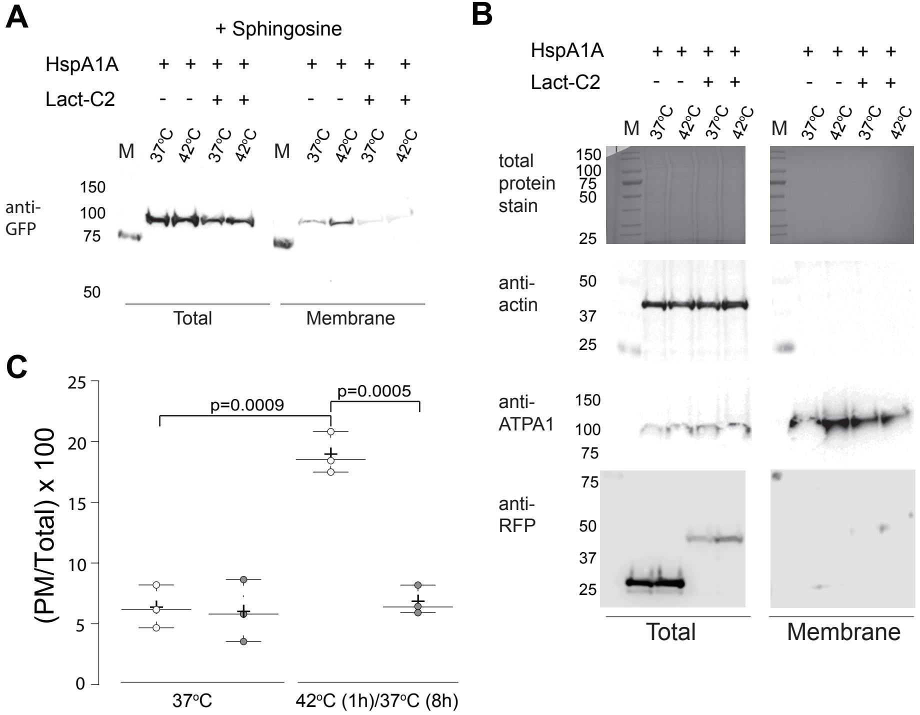

Supplementary Fig. 8
